# Novel Therapeutic Targets in the Brain Tumor Microenvironment

**DOI:** 10.18632/oncotarget.526

**Published:** 2012-05-25

**Authors:** Joanna J. Phillips

**Affiliations:** ^1^ Department of Neurological Surgery, University of California San Francisco; ^2^ Department of Pathology, Division of Neuropathology, University of California San Francisco

**Keywords:** Glioblastoma, proteoglycans, HSPG, sulfs, sulf2, tumor microenvironment

## Abstract

Glioblastoma (GBM), a highly malignant brain tumor of adults and children, diffusely invades within the non-neoplastic brain. Despite aggressive current therapeutic interventions, improved therapeutic strategies are greatly needed. Interactions between the tumor and constituents of its microenvironment are known to regulate malignancy, and heparan sulfate proteoglycans (HSPGs) are important as they bind diverse extracellular proteins, including growth factors and cell adhesion molecules, regulating the activity of several ligand-mediated signaling pathways. Recent work from our group described a mechanism by which GBM regulates PDGFR-alpha signaling via enzymatic alteration of heparan sulfate proteoglycans (HSPGs) in the extracellular microenvironment. Blocking tumor-induced alterations of HSPGs, which can be achieved by pharmacological strategies, would potentially inhibit multiple oncogenic signaling pathways in tumor cells and disrupt critical tumor-microenvironment interactions. Here we examine HSPGs and the enzymes that modify them in GBM. We compare their expression across tumor subtypes, their potential roles in oncogenesis, and their potential as novel therapeutic targets in GBM.

## INTRODUCTION

Primary malignant brain tumors make up one of the deadliest forms of cancer in both adults and children. Glioblastoma (GBM), the most common primary malignant brain tumor in adults, is a highly malignant diffuse astrocytoma that can present *de novo* or develop from the progression of a lower grade tumor. Children also present with GBM, although less commonly. For any age group, GBM share a similar histopathologic appearance between tumors, however GBM are highly heterogeneous with respect to their biologic and molecular characteristics. Using a combination of gene expression, genomic and proteomic data to identify patterns between GBMs, tumors can be stratified into potentially clinically relevant subtypes [1-8]. Future stratification of patients into subgroups based upon predicted responsiveness to specific therapies will likely lead to improved therapy. Despite these exciting advances, the current prognosis for patients with GBM is poor and median survival remains less than two years [9]. There is great need for improvement in our understanding of factors driving tumorigenesis within different tumor subtypes. This knowledge will propel the development of novel therapeutic strategies and advance our ability to target tumors and to predict response.

Aberrant activation of multiple receptor tyrosine kinase (RTK) signaling pathways is a unifying feature across GBM, promoting many aspects of tumorigenesis including tumor cell proliferation, survival, invasion, and induction of angiogenesis. Indeed the most common alterations in GBM include amplification of RTK receptors, such as EGFR and PDGFRA, and increased expression of ligands, such as PDGFB [5, 10-12]. Furthermore, overexpression of ligands such as PDGFB can drive tumorigenesis in murine brain tumor models [13-15]. Despite the role of RTKs in driving oncogenesis, small molecule inhibitors targeting single RTK pathways have been largely unsuccessful in improving overall survival [16]. A potential explanation for the limited efficacy of these targeted therapeutics is that GBM is driven by the summation of multiple signaling inputs [1, 17-19]. Simultaneous targeting of multiple abnormal signaling pathways will likely be required for the development of more effective therapies.

During normal development and tissue maintenance ligand-mediated signaling is exquisitely regulated, including the bioavailability of ligand in the extracellular environment. A prototypical example is the extracellular regulation of the Wnt family of secreted proteins. Once released from the cell Wnt ligands bind and are sequestered by proteins such as heparan sulfate proteoglycans (HSPGs) in the extracellular environment. Only after ligands are released from HSPGs can they bind to and activate their Frizzled receptors [20, 21]. While the mechanisms regulating ligand availability in the tumor microenvironment are just beginning to be elucidated in GBM, they likely play a role in driving oncogenic signaling pathways. Blocking these mechanisms has the potential to inhibit ligand-mediated activation of multiple oncogenic pathways in tumors.

### Heparan sulfate proteoglycans and extracellular sulfatases in GBM

**Figure 1:**
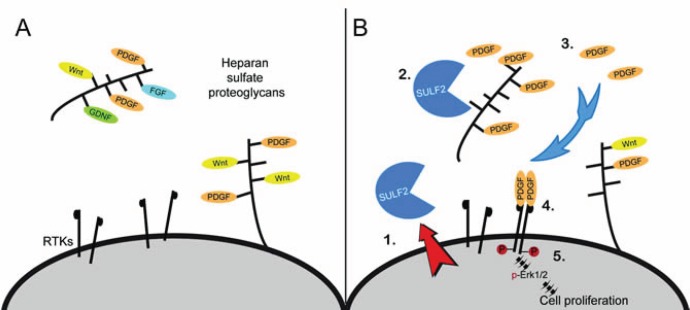
Heparan sulfate proteoglycan (HSPG) glycosaminoglycan side chains bind and sequester ligands in the extracellular environment (A) Dependent on the HSPG core protein, HSPGs are found at the cell surface, in the extracellular matrix, or in secretory vesicles. HSPG function is critical for normal growth and development and includes regulation of ligand-mediated signaling, cell adhesion, and formation of the extracellular matrix for cell migration. (B) Model for SULF2 regulated RTK signaling in glioblastoma. SULF2 acts on HSPGs, present in the tumor microenvironment, to decrease 6O-sulfation, release sequestered ligands such as PDGF and increase activation of the RTK PDGFR-alpha and downstream signaling pathways in tumor cells. RTK, receptor tyrosine kinase.

HSPGs present on the cell surface as well as in the extracellular matrix, are a major component of the extracellular environment in normal brain and GBM [22, 23]. They regulate cellular signaling via their ability to bind diverse protein ligands including growth factors, chemokines, morphogens, matrix proteins, cell adhesion molecules, and proteases [24-28]. As illustrated in Figure 1A, HSPGs can bind and sequester ligands thereby preventing engagement with their cognate receptor, as discussed above with the Wnts for example. HSPG can also act as a co-receptor and promote receptor signaling, such as with FGF2 and VEGF [20, 29, 30]. HSPG-mediated signaling is also critical for normal brain development [31].

**Figure 2:**
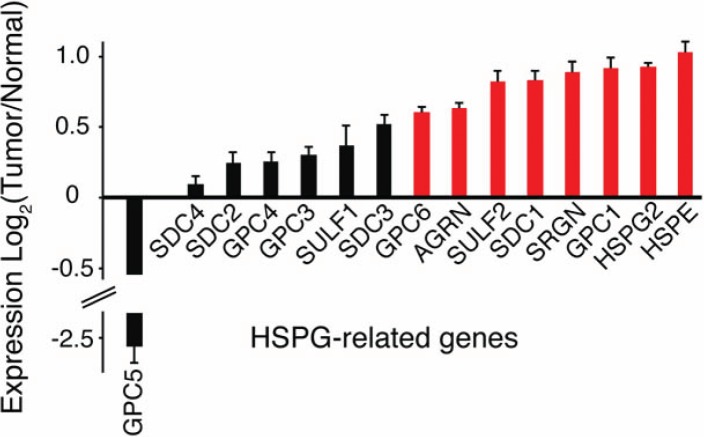
Altered HSPG-related gene expression in human GBM The mean expression of a number of HSPG-related genes, including HSPG core proteins (GPCs, SDCs, AGRN, SRGN, and HSPG2) and modifying enzymes (HPSE and SULFs) are altered in GBM relative to normal controls. Bars represent the mean ratio of log_2_(Tumor/Normal) +/− SEM gene expression. Upregulated (red) and down regulated, log_2_ (Tumor/Normal) greater than or equal to 0.5 or less than or equal to -0.5, respectively. TCGA Data Portal [71]; *http://cancergenome.nih.gov*. (n=170 human tumors). GPC, glypican; SDC, syndecan; SULF, extracellular sulfatase; AGRN, agrin; SRGN, serglycin; HSPG2, perlecan; HSPE, heparanase.

In GBM, the expression levels of multiple HSPG core proteins and HSPG-modifying enzymes are significantly altered relative to normal brain (Figure 2). HSPGs consist of a protein core and heparan sulfate (HS) chains consisting of linear carbohydrate chains of repeating disaccharide units. Essential for their function in cell signaling, the HS chains undergo extensive post-translational modifications, including sulfation on the 6-O- position of glucosamine [32]. Indeed, 6-O-sulfation of HS is a critical determinant of growth factor binding and is essential for normal development [33-37]. While a number of intracellular enzymes regulate HSPG biosynthesis and sulfation, the recently discovered extracellular sulfatases, SULF1 and SULF2, reveal a novel mechanism for the regulation of HSPG-dependent signaling. By removing 6-O-sulfates on HS chains and mobilizing protein ligands from HSPG sequestration in the extracellular environment, the Sulfs can activate multiple key signaling pathways (e.g., Wnt, Shh, GDNF, and PDGF) [20, 29, 38-40].

**Figure 3:**
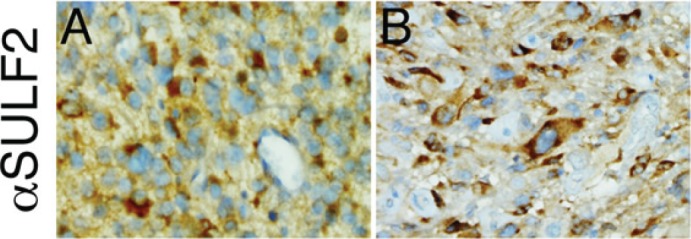
Expression of SULF2 protein in human GBM Representative images of immunohistochemical staining for SULF2 in adult (A) and pediatric (B) GBM. Immunohistochemistry was performed as described previously [40]. Magnification 400x.

**Figure 4:**
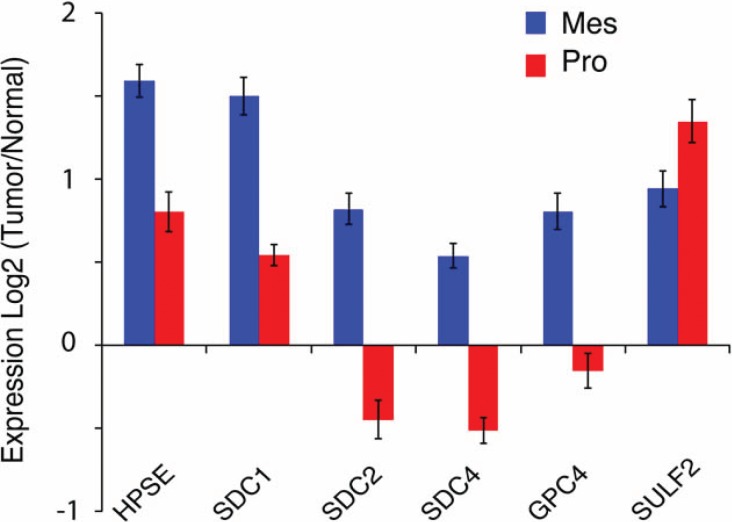
Subtype-specific alterations in the expression of HSPGs and HSPG modifying enzymes in GBM The mean expression of HSPG-related genes are compared between the Mesenchymal (Mes) and Proneural (Pro) subtype of adult GBM. Bars represent the mean ratio of log_2_(Tumor/Normal) +/− SEM gene expression in each subgroup and a two-sided t-test was used to compare expression between the two groups. p<0.05, Mes n=56 and Pro n=53. Expression data from TCGA Data Portal [71]; *http://cancergenome.nih.gov*.

Consistent with this ability, SULF transcripts are overexpressed in GBM and in many human cancers, including non-small cell lung cancer (NSCLC), hepatocellular carcinoma, breast cancer, head and neck cancer, pancreatic adenocarcinoma, multiple myeloma, and gastric carcinoma [40-42]. In GBM, we have found that SULF2 protein is expressed in adult and pediatric tumors (Figure 3) and, using knockdown and transgenic approaches, we have demonstrated ablation of SULF2 results in decreased activity of several RTKs, including PDGFR-alpha, decreased tumor cell proliferation, and prolonged survival *in vivo*[40]. Interestingly, SULF2 has also been directly implicated as a driver of carcinogenesis in NSCLC [43], pancreatic cancer [44], hepatocellular carcinoma [45], and a murine model for oligodendroglioma [46], further supporting its importance. The SULFs appear to regulate multiple signaling pathways important in cancer, likely upstream to the interaction of growth factors with RTKs and the activation of intracellular kinases (Figure 4). Defining the extent and timing of SULF2 function in tumorigenesis will be important.

HSPGs are also the targets of heparanase (HPSE), an endoglycosidase, which generates biologically active fragments of HS chains. Heparanase is upregulated in many cancers, including GBM (Figure 2 and [47-49]). Increased expression of heparanase in tumors has been implicated in increased tumorigenesis, tumor angiogenesis, and invasiveness [47-49]. Together these studies suggest that tumors actively enzymatically modify components of the brain tumor microenvironment to help drive oncogenic signaling and invasion. Disruption of this partnership may be an important therapeutic strategy.

In addition to enzymatic alterations in HSPGs, the identity and levels of the HSPG core proteins are also important determinants of cell signaling. The core protein specifies proteoglycan localization and can influence both extracellular and intracellular signaling. For example, the syndecans (SDCs), composed of four members, are membrane bound HSPGs and contain a cytoplasmic domain that binds cytoskeletal proteins and can serve as a substrate in cell signaling [50, 51]. SDCs play important roles in cell signaling, cell adhesion, and migration. Manipulation of syndecan-1 expression has been shown to alter HGF-Met signaling [52, 53] and Wnt signaling [54] in cancer. In contrast, the glypicans (GPCs) composed of six members, are GPI-linked to the cell membrane, and these are typically involved in growth factor and morphogen responses. Other HSPGs, such as perlecan (HSPG2) are found within the extracellular matrix. For those HSPGs associated with the cell membrane, the extracellular domain can also be shed, resulting in the release of biologically active proteoglycans. Indeed, shed syndecan-1 has been shown to mediate removal of CXC chemokines and facilitate resolution of inflammation [55]. In multiple myeloma high levels of shed syndecan-1 correlate with poor prognosis and have been associated with increased tumor growth in animal models [56, 57]. In GBM, both total and specific HSPGs core proteins are altered (Figure 2), including increased levels of syndecan-1 and glypican-1 as previously demonstrated [23, 58].

Interestingly, alterations in HSPGs vary across tumor subtypes suggesting there may be subtype-specific HSPG functions in GBM, Figure 4. The proneural GBM subtype, characterized by alterations in PDGFR signaling, has high SULF2 expression [4]. In contrast, the mesenchymal GBM subtype exhibits increased expression of multiple other HSPG-related genes. This latter subtype has increased expression of genes involved in interactions with the extracellular environment, cell signaling, and the immune response, and, consistent with gene expression data, the number of tumor-associated microglia/macrophages is significantly greater in the mesenchymal compared to the proneural subtype (Mann-Whitney, p=0.042; J. Engler and J. Phillips, unpublished observation). While the mechanisms driving the increased inflammatory response in the mesenchymal subtype are not fully elucidated, proteoglycans have the potential to influence the immune response in cancer [59]. Indeed, tumor-derived versican has been shown to activate macrophages and increase metastatic tumor growth in a model for lung carcinoma [60]. Furthermore, targeting of HSPGs with a heparan sulfate (HS) mimetic normalized myeloid-derived suppressor cell levels in a murine mammary carcinoma model [61]. Understanding the function of HSPGs and the enzymes that modify them in a subtype-specific context will be important for the optimization of future therapeutic strategies. This has recently been illustrated by the report of an immunogenic therapy in which patients with a GBM of the mesenchymal subtype had a more robust immune response than patients with a tumor of the proneural subtype [62].

### HSPGs and the enzymes that regulate them as potential therapeutic targets in GBM

Accumulating data suggest the extracellular HSPGs, and the enzymes that modify them, may regulate ligand-mediated signaling pathways in GBM. Given their role in disease combined with their accessibility in the extracellular environment, they represent clinically relevant, druggable therapeutic targets. In malignant astrocytoma, we recently determined that knockdown of SULF2 resulted in decreased activity of multiple RTK signaling pathways including PDGFR-alpha, IGF1R-beta and EPHA2 [40], three pathways known to be involved in astrocytoma growth and invasion [10, 13, 14, 63-65]. Furthermore, ablation of SULF2, in a relevant murine model for astrocytoma, resulted in decreased activation of PDGFR-alpha, decreased tumor cell proliferation, and prolonged survival [40]. These data, combined with the high expression of SULF2 in a significant number of human GBMs, suggest SULF2 may be considered an upstream therapeutic target in the treatment of GBM and other cancers in which it is overexpressed.

Since HSPGs regulate multiple upstream signaling pathways, and some of these same pathways are critical in malignancy, it is of great interest that a recent class of compounds has been developed to inhibit some of these oncogenic functions. Heparan sulfate mimetics are highly sulfated oligosaccharides that inhibit heparanase, sequester HSPG-binding factors, and inhibit SULF2 [66-68]. In preclinical studies, HS mimetics have effectively targeted multiple HSPG-dependent phenotypes and have resulted in decreased in vivo tumor growth, tumor invasion, tumor metastasis, and angiogenesis [61, 69]. Furthermore, a human Phase II clinical trial demonstrated safety and preliminary efficacy for a HS mimetic in recurrent hepatocellular carcinoma [70], and a recent preclinical study of a new rationally engineered HS mimetic, M402, suggests additional potential as a therapeutic agent [61]. While HS mimetics have not yet been tested in GBM, they are known to inhibit SULF2 activity [67] and represent a promising strategy.

Currently the prognosis for patients with GBM is challenging. With recent advances in imaging, genomic sequencing and proteomics, there is great hope that we are entering into a new era for detection and treatment of GBM. Stratification of patients into therapeutically relevant subgroups will likely be an essential component for treatment. Large-scale analyses of bulk tumors have revealed significant differences in expression of genes involved in tumor-microenvironment interactions between tumor subgroups, including proteoglycans and immune response-related gene. Targeting HSPGs and related components of the tumor microenvironment has the potential to simultaneously inhibit multiple oncogenic signaling pathways in tumor cells and to disrupt critical tumor-microenvironment interactions. Future efforts will be aimed at identifying the relevant tumor-microenvironment interactions that help drive GBM and how to effectively target them therapeutically.
